# Biosensor Based on Peroxidase-Mimetic Nanozyme and Lactate Oxidase for Accurate L-Lactate Analysis in Beverages

**DOI:** 10.3390/bios12111042

**Published:** 2022-11-18

**Authors:** Oleh Smutok, Taras Kavetskyy, Tetiana Prokopiv, Roman Serkiz, Ondrej Šauša, Ivan Novák, Helena Švajdlenková, Igor Maťko, Mykhailo Gonchar, Evgeny Katz

**Affiliations:** 1 Department of Chemistry and Biomolecular Science, Clarkson University, Potsdam, NY 13699-5810, USA; 2 Department of Biology and Chemistry, Drohobych Ivan Franko State Pedagogical University, 82100 Drohobych, Ukraine; 3 Department of Materials Engineering, The John Paul II Catholic University of Lublin, 20-950 Lublin, Poland; 4 Institute of Cell Biology, National Academy of Sciences of Ukraine, 79005 Lviv, Ukraine; 5 Department of Nuclear Chemistry, Comenius University in Bratislava, 84215 Bratislava, Slovakia; 6 Institute of Physics, Slovak Academy of Sciences, 84511 Bratislava, Slovakia; 7 Polymer Institute, Slovak Academy of Sciences, 84541 Bratislava, Slovakia

**Keywords:** peroxidase-mimetic, nanozyme, electrochemical biosensor, L-lactate, beverages, analysis

## Abstract

Precision analysis of the key biological metabolites such as L-lactate has great practical importance for many technological processes in food technology, including beverage production. Here we describe a new, highly selective, and sensitive biosensor for accurate L-lactate assay based on a combination of peroxidase-mimetic nanozymes with microbial lactate oxidase (LOx) immobilized onto the surface of a graphite-rod electrode (GE). The peroxidase-like nanozymes were synthesized using the debris of carbon microfibers (CFs) functionalized with hemin (H) and modified with gold nanoparticles (AuNPs) or platinum microparticles (PtMPs). The nanozyme formed with PtMPs as well as corresponding bioelectrodes based on it (LOx-CF-H-PtMPs/GE) is characterized by preferable catalytic and operational characteristics, so it was selected for the analysis of L-lactate content in real samples of grape must and red wine. The results of the L-lactate analysis obtained by the developed biosensors are highly correlated with a very selective spectrophotometric approach used as a reference. The developed biosensor, due to its high selectivity and sensitivity, is very prospective not only for the beverage industry and food technology, but also for clinical diagnostics and medicine, as well as in other applications where the accurate analysis of L-lactate is highly important.

## 1. Introduction

L-lactate (or L-lactic acid) is a normal physiological metabolite of almost all living organisms from microbial to human beings. It is produced also on a high scale during microbial fermentation in different technological processes, including beverage production, affecting the final product quality and its storage properties. The L-lactic acid is produced from malic acid (naturally present in grape must) during secondary microbial malolactic fermentation in winemaking. Natural L-lactic acid has a softer taste compared to malic acid, which is tart-tasting [1]. The ratio between both acids is a strong marker of red wine quality [2] and is used for certification of very expensive vintage wines. Moreover, as an effective pH-conservator L-lactate itself stabilizes the wine, preventing bacterial growth and eliminating the risk of spoilage, production of undesirable flavors, and color changes during storage [1]. Accordingly, its analysis in the beverage industry has great importance for indicating the quality and storage properties of natural wine.

There are many L-lactate selective electrochemical biosensors described recently. Most of them are based on the usage of three enzymes, one of mammalian origin—NAD^+^-dependent lactate dehydrogenase (EC: 1.1.1.27, LDH) [3,4] and two microbially produced: L-lactate: cytochrome *c* oxidoreductase (EC: 1.1.2.3, flavocytochrome *b*_2_, FC *b*_2_) [5,6,7] and lactate oxidase (EC: 1.13.12.4, LOx) [8,9,10]. Usually, the enzymes are used separately, but sometimes LOx and LDH could be combined into one biosensing element [11]. LOx biosensors are based on the principle of direct electrochemical detection of H_2_O_2_ which is a coproduct of enzymatic reaction [12,13] or indirectly by means of its detection through an additional enzyme—horseradish peroxidase (EC 1.11.1.7, HRP) [14]. As a result of the introduction of HRP into the LOx-based biosensor system, a significant decrease in the working potential of the sensor is achieved due to the ability of HRP to provide an electron transfer at low working potentials. This significantly improves the selectivity of the biosensor, but simultaneously it complicates the procedure for preparing working electrodes and reduces their stability [14]. Hence, as can be seen, the usage of natural enzymes, especially using more than one simultaneously, in biosensor technology has some drawbacks resulting in limited stability and high cost of the biosensors. However, due to significant progress in materials science and nanotechnology, nowadays there is an open possibility to use synthesized artificial enzymes (nanozymes) instead of natural proteins. In contrast to natural enzymes, the nanozymes usually are less catalytically active. However, they are very stable in the surrounding environment, lower in cost, and easy to recover and store. Moreover, they have a large surface area suitable for chemical modification and are often highly conductive [15]. All these properties make them perspective candidates as nonbiological recognition elements in electrochemical biosensors. The first nanozyme was described back in 1970 by Breslow et al. [16]. From that time, hundred different types, structures, and properties nanozymes were synthesized, characterized, and successfully used in sensor technology [17,18,19,20]. Despite the large number of lactate biosensors created on the basis of different nanocomposites, increasing their sensitivity and their adaptation for the analysis of the target analyte in complex mixtures of real samples is still an urgent task. The purpose of this study is devoted to this problem.

In this paper, we describe the synthesis and application in sensor technology of the peroxidase-mimetic nanozymes in combination with a new conductive micro/nanocomposite. The micro/nanocomposites were based on the debris of carbon microfibers functionalized with hemin and modified with noble metals (gold or platinum) micro- or nanoparticles. It was demonstrated that platinum-based microcomposite is characterized by improved catalytic parameters compared to gold-based analog; hence, in combination with lactate oxidase, it was chosen for the construction of the L-lactate selective biosensor for beverage analysis.

## 2. Materials and Methods

### 2.1. Materials

Delignified cellulose (Greencel) was obtained from Bukoza and was used without any additional pre-treatment. L-lactate oxidase (EC 1.1.3.2, LOx) from *Pediococcus* sp. with specific activity ≥ 20 U·mg^−1^ solid, Nafion^®^, hemin, guaiacol, hydrazine sulfate, L-lactate, HAuCl_4_, and H_2_PtCl_6_ were obtained from Sigma-Aldrich. In addition, 28% ammonia solution, Na_2_HPO_4_, CaCl_2_, KН_2_РО_4_, HNO_3_, and sodium citrate were purchased from Merck, and 30% H_2_O_2_ from Thermo Fisher Scientific. All reagents and solutions were prepared using ultrapure water.

### 2.2. Synthesis and Functionalization of Carbon Microfibers

Carbon microfibers (CFs) were prepared by carbonization of delignified cellulose at 700 °C without air access using Elektro oven (Bad Frankenhausen), then boiled for 1 h in 65% HNO_3_, washed out from acid, and dried [21,22].

Functionalization of CFs with hemin was carried out according to the published method [23,24]. A total of 5.0 mL (0.5 mg·mL^−1^) of CF suspension was sonicated for 3 min to obtain a homogenous suspension of micro-sized debris. Then, the formed suspension of the CF debris was mixed with 5.0 mL of hemin solution (0.5 mg·mL^−1^) and 50 μL of ammonia (14.76 M), after which 10 μL of hydrazine solution (15.6 M) was added. After intensive shaking for several minutes, the sample was placed in a thermal bath (60 °C) for 4 h. The obtained black dispersion (CF-H) was washed twice with water and centrifuged for 15 min at 9700 g using Hettich Micro-22R centrifuge.

Modification of CF-H by gold (Au^0^) or platinum (Pt^0^) was carried out by a reduction in noble metals from the appropriate acids (HAuCl_4_ or H_2_PtCl_6_) using wet chemistry according to the published method [25]. Moreover, 1 mL CF-H resuspended in water (0.5 mg·mL^−1^) was premixed with 1 mL of HAuCl_4_ (0.3 mM) or 1 mL of H_2_PtCl_6_ (0.3 mM), after which 1 mL sodium citrate (1.5 mM) was added. To remove excess reagents, the CF-H-AuNPs and CF-H-PtMPs preparations were washed twice with water followed by centrifugation for 30 min at 9700 g, then resuspended in water to a final concentration of 2.5 mg·mL^−1^ and stored at 4 °С until use.

### 2.3. Estimation of Peroxidase Activity of Synthesized Nanozymes

Estimation of peroxidase-like activity of the synthesized CF-H-AuNPs and CF-H-PtMPs was performed using UV-VIS-spectrophotometry on Shimadzu UV-1650 PC. The reaction solution includes 25 mM guaiacol (a chromogen) and 8.8 mM H_2_O_2_ in 25 mM MES buffer, pH 5.5 at 50 °C [26].

### 2.4. Scanning Electron Microscopy and X-ray Microanalysis

A Scanning Electron Microscope (SEM-microanalyser REMMA-102-02, Sumy) was used for structural and morphological analysis of the CF-H-AuNPs and CF-H-PtMPs. The micro/nanocomposites were immersed in a Butvar solution B-98 (Sigma-Aldrich) in 1.5% chloroform and ultrasonicated. All the used parameters, WD—distance from the last lens of a microscope to the samples (mm); kV—accelerating voltage; х—magnification fold; and scale unit (μm), are indicated in the corresponding SEM microphotographs.

### 2.5. Electrochemical Analysis

The electrochemical measurements were carried out in a three-electrode configuration, including a graphite-rod working electrode, a Pt wire as a counter electrode, and Ag/AgCl/KCl (3M) reference electrode. The 3.05 mm graphite rods (type RW001, from Ringsdorff Werke) inserted and fixed in a glass tube by epoxy glue were used as working electrodes. Before usage, the working electrodes were polished with emery paper (P2000 Smirdex^®^). The amperometric measurements were performed in a standard electrochemical cell using a potentiostat CHI 1200A (IJ Cambria Scientific, Llanelli, UK) at room temperature under continuous stirring in a magnetic field.

### 2.6. Preparation of L-Lactate Selective Bioelectrodes

To modify the working electrodes by nanozymes, 3 µL aqueous suspension (0.5 mg·mL^−1^) of CF-H-AuNPs or CF-H-PtMPs was dropped on the electrode surface, followed by drying for 15 min at room temperature. Then, 5 µL L-lactate oxidase solution (with the activity of 200 U·mL^−1^) was dropped on the top of the same working electrode. The formed bio/microcomposite layer was fixed with 5 µL 1% neutralized aqueous solution of Nafion^®^ and kept for 15 min at room temperature till fully dry. The ready-to-use LOx/microcomposite electrodes were rinsed twice with 50 mM phosphate buffer (PB), рН 6.8, and kept in a glass exicator at 4 °C till usage.

### 2.7. Preparation and Analysis of Real Samples

A grape must and dry red wine “Isabella” (Ukraine) was used for the evaluation of the accuracy of the developed L-lactate selective biosensor. The samples were stored at −20 °C till testing. The grape must was diluted (the final dilution) with 50 mM PB, рН 6.8 in 200-fold, and wine in 400-fold, respectively. Any additional treatment of the samples except their dilution was omitted. A “standard addition analysis”, as a proven approach for precision analysis of complex biological solutions [27], was used.

## 3. Results and Discussion

**Figure 1:**
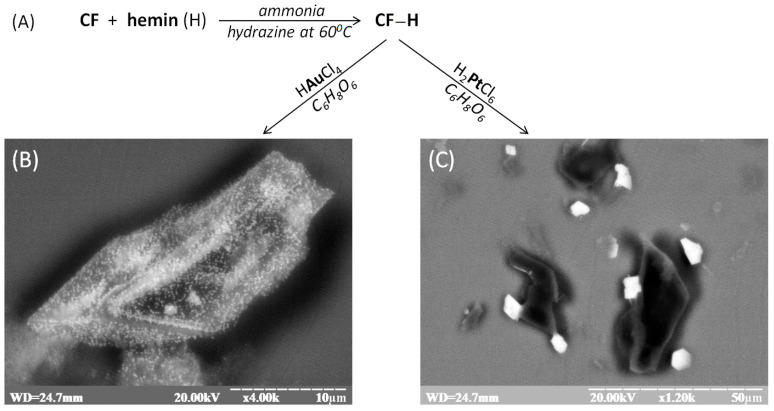
The principal scheme of synthesis of peroxidase-mimetic nanozymes based on the debris of CF modified by hemin (H) in combination with noble metal micro- or nanoparticles (**A**). SEM images of the obtained catalytic composites with incorporated AuNPs (**B**) and PtMPs (**C**). Abbreviations: WD—distance from the last lens of a microscope to the samples (mm); kV—accelerating voltage; х—magnification fold; μm—scale unit.

As a basis for the synthesis of efficient peroxidase-mimetic nanozymes, the debris of carbon microfibers (CFs) were used. The CF was prepared according to the previously described procedure [21,22]. For the nanozymes synthesis developed by us recently, a procedure with some modification was proposed [24]. It includes the functionalization of CF by hemin (compound characterized with a small peroxidase activity) in combination with gold nanoparticles (AuNPs) or platinum microparticles (PtMPs) (Figure 1 and Figure S1).

Scanning Electron Microscopy (SEM) demonstrated the formation of Au-nanoparticles (Figure 1B) and Pt- microparticles (Figure 1C) on the hemin-functionalized CF surface. To confirm the results, X-ray spectral analysis was used (Figure S2). The X-ray spectra confirmed the formation of different structural types of Au^0^, with the typical K_α_ peaks at 2.15; 9.75; 11.5 keV (Figure S2B) and the presence of Pt^0^ with K_α_ peaks at 2.1; 9.45, and 11.2 keV for the CF debris modified by hemin (Figure S2C).

**Figure 2:**
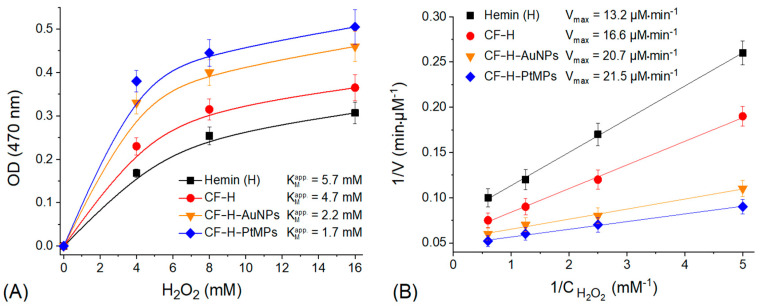
Dependence of the rate of the peroxidase-like reaction on the concentration of the substrate (H_2_O_2_) in solution to determine the Michaelis–Menten constant of CF-H-AuNPs and CF-H-PtMPs nanozymes in comparison with hemin and CF-H as controls (**A**). Analysis of specific peroxidase activity of CF-H-AuNPs and CF-H-PtMPs nanozymes compared to the same controls (**B**). Conditions: initial concentration of the nanozymes, hemin and CF-H 1.0 mg·ml^−1^; 25 mM MES buffer, pH 5.5; 25 mM guaiacol; reaction time 30 min at 60 °C. Abbreviations: K_M_^app^—apparent Michaelis–Menten constant; V_max_—maximal rate of the reaction.

The modification of the hemin-functionalized CF by AuNPs (CF-H-AuNPs) or PtMPs (CF-H-PtMPs) facilitates the electron transfer from H_2_O_2_ to hemin when the electroactive debris of CF play a binary function: as support, binding micro/nanoparticles of noble metals and hemin, and as a transducer, providing the efficient electron-transfer communication between the electrode surface and hydrogen peroxide. As there was no observed peroxidase activity of the nanocomposites without their modification by hemin, exactly hemin is the main source of the activity. The main difference between nanozymes and electrocatalysts is their ability to specific catalysis in a solution without applied external potentials. The catalytic characteristics of the synthesized peroxidase-mimic nanozymes (CF-H-AuNPs and CF-H-PtMPs) were tested via estimation of their specific peroxidase activity (V_max_) and the apparent Michaelis–Menten constant (K_M_^app^) toward H_2_O_2_ in comparison with hemin and hemin-functionalized CF (CF-H) as controls (Figure 2).

It was demonstrated that the affinity toward H_2_O_2_ for both types of synthesized nanozymes (CF-H-AuNPs and CF-H-PtMPs) was significantly increased compared with hemin in solution. The calculated value of K_M_^app^ for CF-H-AuNPs decreased 2.6-fold (2.2 mM vs. 5.7 mM) when K_M_^app^ for CF-H-PtMPs decreased even up to 3.4-fold (1.7 mM vs. 5.7 mM) (Figure 2A). The peroxidase-like activity of the micro/nanocomposites CF-H-AuNPs and CF-H-PtMPs is about 1.6-fold higher compared with the activity of hemin in solution as a control (2.07 and 21.5 µmol·mg^−1^·min^−1^ vs. 1.32 µmol·mg^−1^·min^−1^, respectively). Some impact of CF on nanozyme peroxidase’s activity was also observed as for the CF-H sample, specific peroxidase activity increased 1.3-fold versus hemin itself (1.66 µmol·mg^−1^·min^−1^ vs. 1.32 µmol·mg^−1^·min^−1^) (Figure 2B). The high specific peroxidase activity of CF-H-AuNPs and CF-H-PtMPs, as well as the high affinity toward hydrogen peroxide, opens the possibility of their using as nanozymes for the construction of an amperometric biosensor based on lactate oxidase (LOx) producing H_2_O_2_ as a coproduct of L-lactated oxidation.

To estimate an optimal working potential to support an efficient electron transfer from the surface of the rod graphite electrode to nanozymes catalyzing peroxide reduction, cyclic voltammetry in the presence of 5 mM H_2_O_2_ was performed (Figure S3). A total of 1% Nafion was used for fixation of sensitive layers on the graphite electrodes surface. It was demonstrated that at working potential −0.1 V vs. Ag/AgCl/KCl (3M), a typical H_2_O_2_ reduction curve is observed as a result of catalysis by CF-H-AuNPs and CF-H-PtMPs-modified electrodes. Notably, this potential is sufficient for the efficient H_2_O_2_ reduction by the immobilized nanozymes and is low enough to prevent possible nonselective impact from easily reducing reagents presented in real beverage samples; hence, it was chosen as a working potential for chronoamperometric analysis.

**Figure 3:**
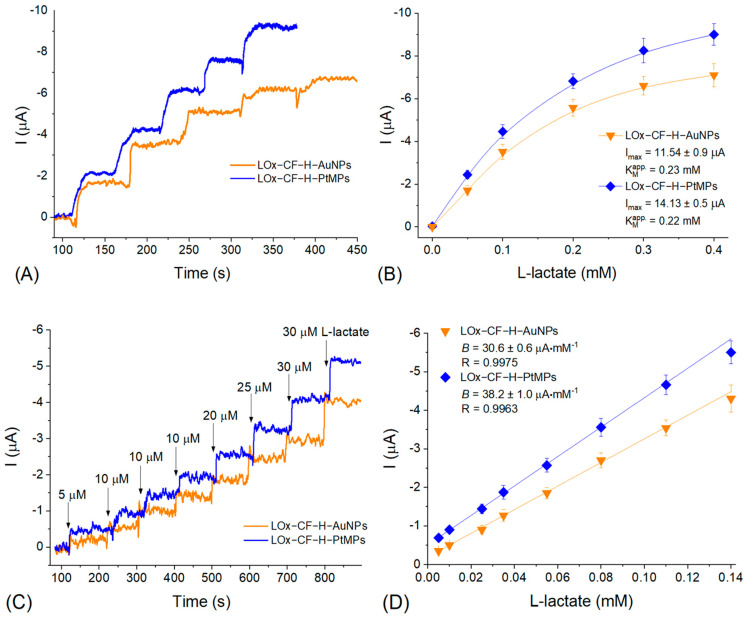
The typical chronoamperometric responses of LOx-CF-H-AuNPs and LOx-CF-H-PtMPs-formed bioelectrodes upon subsequent additions of increasing L-lactate concentrations (**A**) and corresponding calibration curves (**B**); the typical chronoamperometric response of the analog electrodes taken in the linear detection frames (**C**) and corresponding calibration in the linear frames (**D**). Conditions: −0.1 V vs. Ag/AgCl/KCl (3M); 50 mM PB, рН 6.8 with 100 mM Na_2_SO_4_; room temperature; continuous stirring. Abbreviations: I_max_—maximal current at substrate saturation; K_M_^app^—apparent Michaelis–Menten constant; *B*—a slope of the curve; R—correlation coefficient for linear regression.

**Figure 4:**
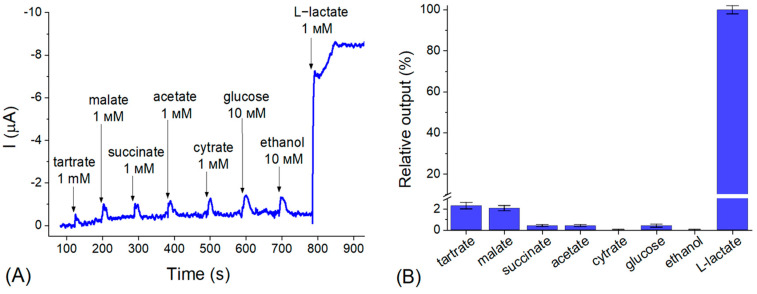
The typical chronoamperometric response (**A**) and the level of relative output (**B**) for LOx-CF-H-PtMPs bioelectrodes toward different metabolites and target analyte. Conditions: −0.1 V vs. Ag/AgCl/KCl (3M); 50 mM PB, рН 6.8 with 100 mM Na_2_SO_4_; room temperature; continuous stirring.

The L-lactate selective electrodes were prepared with the combination of nanozymes (CF-H-AuNPs and CF-H-PtMPs) with LOx as described above. In respect of the estimation of the main operational parameters of the formed bioelectrodes (LOx-CF-H-AuNPs and LOx-CF-H-PtMPs), they were tested using the chronamperometric method (Figure 3 and Figure 4).

The typical calibration curves for L-lactate derived from the corresponding chronoamperograms of the constructed bioelectrodes based on two nanozymes are presented in Figure 3. The LOx-CF-H-AuNPs bioelectrodes were characterized by a value of maximal current at substrate saturation (I_max_) as 11.54 ± 0.9 µA, and the apparent Michaelis–Menten constant (K_M_^app^) of 0.23 mM L-lactate. The value of I_max_ for the bioelectrodes based on another nanozyme (CF-H-PtMPs) is estimated as 14.13 ± 0.5 µA and the K_M_^app^ value is equal to 0.22 mM L-lactate (Figure 3A). The saturation of the sensor output at the increased concentrations of L-lactate depends on the catalytic properties of the recognition elements (most on LOx and less on nanozyme) when excessive reaction products begin to occupy their catalytic sites [28]. The limit of detection (LOD) for both types of bioelectrodes was calculated as 2 µM, and the linear frames were from 5 µM to 0.14 mM L-lactate (Figure 3D). The sensitivity of the bioelectrodes was calculated from the slope of the calibration curves in the linear frames (*B*) considering a working area (geometric area) of the electrode used (7.3 mm^2^). The estimated sensitivity for the LOx-CF-H-AuNPs bioelectrodes was calculated as 4191.8 A·M^−1^·m^−2^ and for the LOx-CF-H-PtMPs it is characterized even by a higher sensitivity 5232.9 A·M^−1^·m^−2^. As such, the LOx-CF-H-PtMPs bioelectrodes were characterized with preferable operational parameters compared to the LOx-CF-H-AuNPs one, and they were chosen for the real samples testing. However, before analyzing the L-lactate content in the real beverages, it is critically important to check the sensor selectivity toward typical metabolites usually presented in the real must and wine samples (Figure 4).

The study of the selectivity of the bioelectrodes toward different possible interferents demonstrated that only tartrate and malate in 1 mM concentration give an insignificant interfering output of about 2% (Figure 4B). Thus, their possible total impact on the determination of the target analyte (L-lactate) in the real samples does not exceed 5%. Taking into account that, due to the very high sensitivity of the sensor, the real beverage samples should be diluted by hundreds-fold, the impact of the listed interferents is negligible, which indicates a high selectivity of the developed L-lactate-selective biosensor.

**Figure 5:**
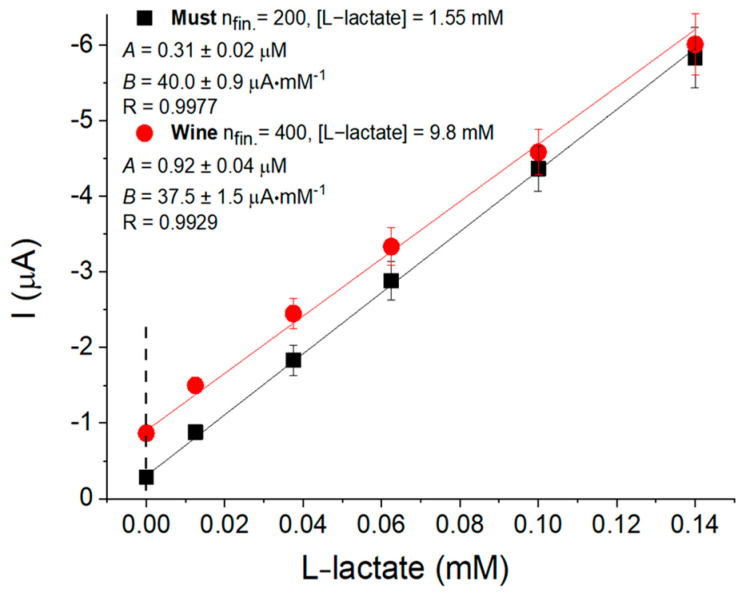
Biosensor analysis of L-lactate content in the real samples of grape must and dry red wine “Isabella” using the “standard addition analysis”. Conditions for biosensor assay: −0.1 V vs. Ag/AgCl/KCl (3M); 50 mM PB, рН 6.8 with 100 mM Na_2_SO_4_; room temperature; continuous stirring. Abbreviations: n_fin._—dilution step; *A*—sensor output to the tested sample; *B*—a slope of the curve; R—correlation coefficient for linear regression.

The constructed LOx-CF-H-PtMPs/GE biosensor was tested for analysis of L-lactate in the grape must and dry red wine “Isabella”. The real samples analysis was performed using the “standard addition analysis” (Figure 5 and the obtained results were compared with results acquired with the help of a highly selective spectrophotometric enzymatic method [29] (Table 1).

The biosensor analysis of L-lactate content in grape must and dry red wine using LOx-CF-H-PtMPs bioelectrodes showed the following results: 1.55 ± 0.04 mM (0.14 g·L^−1^) in grape must and 9.76 ± 0.3 mM (0.88 g·L^−1^) of L-lactate in wine (Figure 5A). The reference analysis of L-lactate in the tested beverages was performed using a spectrophotometric approach based on a highly selective L-lactate: cytochrome *c* oxidoreductase (EC: 1.1.2.3) (Figure 5 inset Table 1). The correlation analysis between the results of determining the content of L-lactate in real beverages by different approaches was estimated as R = 0.999, which indicates a very high accuracy and reliability of the analysis by the developed biosensor.

It should be noted that the procedure of the synthesis and application of peroxidase-mimic nanozyme (based on CF, hemin, and AuNPs) in sensor technology was described by us recently [24]. This nanozyme is used in the current work only as the “gold standard” to show improvements in sensor characteristics using PtMPs-based nanozyme compared to AuNPs-based ones. We believe that decreasing the PtMPs size to a nanoscale should improve its catalytic and electron-transfer properties even more significantly, and research in this direction is underway.

The main operational characteristics of the developed L-lactate selective biosensor (LOx-CF-H-PtMPs/GE) were compared with the parameters of recently described analogs (Table 2).

The developed L-lactate selective biosensor is characterized by a 2.8–3.3-fold higher sensitivity in comparison to the most sensitive similar biosensors [9,10] (Table 2). Moreover, the main drawback of most similar biosensors is the application of the high working potentials (+0.3–0.6 V vs. Ag/AgCl) that negatively affect their selectivity. It should be noted that some organic compounds of the real samples are capable of co-oxidizing at the above-mentioned potentials (e.g., ascorbic, succinic, citric acids, neurotransmitters, pigments, drugs, and even glucose) resulting in a decrease in assay accuracy. On the other hand, when the usage of the working potential is close to zero volts (vs. Ag/AgCl), this does not decrease the sensor selectivity.

It can be assumed that the developed biosensor, due to its high selectivity and sensitivity, may be well applicable not only in food technologies but also in clinical diagnostics and medicine, as well as in other branches of analytical biochemistry where the accurate analysis of L-lactate is very important.

## 4. Conclusions

A new L-lactate selective biosensor based on a combination of peroxidase-mimetic nanozyme and microbial lactate oxidase (LOx) was constructed and characterized. Two different nanozymes were used in the work. Nanozymes were synthesized using the debris of carbon microfibers (CFs) functionalized with hemin (H) and modified with gold nanoparticles (AuNPs) or platinum microparticles (PtMPs). The nanozyme formed by PtMPs as well as corresponding bioelectrodes (LOx-CF-H-PtMPs/GE) were characterized by preferable catalytic and operational characteristics so they were tested on the real samples of grape must and dry wine. The high accuracy of biosensor analysis was approved using a highly selective enzymatic approach as a reference. The developed biosensor is characterized by improved selectivity and a 2.8–3.2-fold higher sensitivity in comparison with the nearest recently described analogs; therefore, it can be proposed for practical usage in food technologies, clinical diagnostics, and medicine.

## Figures and Tables

**Table 1:** Comparison of results of L-lactate assay in the real samples obtained by the biosensor and enzymatic analysis.

Sample	Methods	
	*Biosensor*	*Enzymatic*
Must	1.55 ± 0.04	1.55 ± 0.35
Wine	9.8 ± 0.3	10.4 ± 0.9
	R = 0.999	

**Table 2:** Comparison of the main operational characteristics of recently developed L-lactate selective amperometric biosensors.

Sensing Element/Electrode	Sample	Wp, V	LOD, µM	Linearity, mM	Sensitivity, A·M^−1^·m^−2^	Ref.
GA-LDH-AuNPs-ERGO-PAH/SPE	wine yogurt	+0.5	1.0	0.5–3.0 4.0–16.0	108.0 28.0	[3]
LDH-CS-Fc/SPCE	serum	+0.38	9.1	0.05–10.0	N/A	[4]
FC*b*_2_-AuNPs/SPGE	saliva sweat	+0.25	100	0.3–2.0	106.0	[5]
Lox-3,4DHS-AuNPs/SPCE	wine beer yogurt	+0.3	2.6	up to 0.8	406.1	[8]
Lox-Cu-MOF-CS-/Pt-SPCE	wines saliva sweat	+0.15	0.75	0.00075–1.0	1627.7	[9]
PB-LOx-CS-AuNWs/TE	sweat juice	−0.1	137	5.0–30.0	1913.0	[10]
Lox-BSA-GlOH-GA/PtE	N/A	+0.6	3.0	0.005–0.4	1150.0	[12]
Lox-Pt&PdNPs/SPCE	wines	+0.6	0.1	0.05–0.8	300.0	[13]
Lox-CF-H-PtMPs/GE	must wine	−0.1	2.0	0.005–0.14	5232.9	this work

Abbreviations: Wp—working potential vs. Ag/AgCl reference electrode; LOD—limit of detection (3δ). Bioelectrodes description**:** GA-LDH-AuNPs-ERGO-PAH/SPCE—glutaraldehyde (GA), NAD^+^-dependent lactate dehydrogenase (LDH), gold nanoparticles (AuNPs), electrochemically reduced graphene oxide (ERGO), poly (allylamine hydrochloride) (PAH)-modified screen-printed carbon electrodes (SPCE); LDH-CS-Fc/SPCE—NAD^+^-dependent lactate dehydrogenase (LDH), chitosan (CS), ferrocene (Fc)-modified screen-print carbon electrode (SPCE); FC*b*_2_-AuNPs/PGE—flavocytochrome *b*_2_ (FC*b*_2_) and gold nanoparticle (AuNPs)-modified planar gold electrode (PGE); LOx-3,4DHS-AuNPs/SPCE—lactate oxidase (LOx), N,N’-Bis(3,4-dihydroxybenzylidene)-1,2-diaminobenzene Schiff base tetradentate ligand-modified gold nanoparticles (3,4DHS-AuNPs) deposited onto screen-printed carbon electrodes (SPCE); LOx-Cu-MOF-CS-/Pt-SPCE—lactate oxidase (LOx), copper metallic framework (Cu-MOF), chitosan (CS), Pt coated screen-printed carbon electrode (Pt-SPCE); PB-LOx-CS-AuNWs/TE—Prussian blue (PB), chitosan (CS), gold nanowires (AuNWs) modified with lactate oxidase (LOx) forming textile electrodes (TE); LOx-BSA-GlOH-GA/PtE—lactate oxidase (LOx), bovine serum albumin (BSA), glycerol (GlOH), glutaraldehyde (GA)-modified platinum wire electrode (PtE); LOx-Pt&PdNPs/SPCE—lactate oxidase (LOx), platinum and palladium nanoparticle (Pt&PdNPs)-modified screen-printed carbon electrodes (SPCE); LOx-CF-H-PtMPs/GE—hemin-functionalized (H) carbon fibers (CFs) modified by Pt-microparticles (PtMPs) and lactate oxidase (LOx) immobilized on graphite electrode (GE).

## Supplementary Materials

The following supporting information can be downloaded at: https://www.mdpi.com/article/10.3390/bios12111042/s1, Figure S1. The schematic figure illustrates the research concept. Figure S2. X-ray spectral analysis of non-modified debris of CF, CF-H-AuNPs, and CF-H-PtMPs. Figure S3. Cyclic voltamperogramms of bare graphite electrode, CF-H-AuNPs-modified, and CF-H-PtMPs-modified electrodes at addition of 5 mM H_2_O_2_.
