# Supplementary material for: Biosensor Based on Peroxidase-Mimetic Nanozyme and Lactate Oxidase for Accurate L-Lactate Analysis in Beverages
Source: Biosensors (Basel). 2022 Nov 18;12(11):1042. doi: 10.3390/bios12111042 (PMC9688602; doi:10.3390/bios12111042)
Supplement: Supplementary file 1 [file biosensors-12-01042-s001.zip › biosensors-1988131-supplementary.pdf]

biosensors MDPI

Supplementary Materials

Biosensor Based on Peroxidase-Mimetic Nanozyme and Lactate Oxidase for Accurate L-Lactate Analysis in Beverages

Oleh Smutok[1]*, Taras Kavetskyy[2,3], Tetiana Prokopiv[4], Roman Serkiz[4], Ondrej Šauša[5,6], Ivan Novák[7], Helena Švajdlenková[7], Igor Maťko[6], Mykhailo Gonchar[2,4], Evgeny Katz[1]*

[1] *Department of Chemistry and Biomolecular Science, Clarkson University, Potsdam, 13699-5810, New York, USA*

[2] *Department of Biology and Chemistry, Drohobych Ivan Franko State Pedagogical University, 82100 Drohobych, Ukraine*

[3] *Department of Materials Engineering, The John Paul II Catholic University of Lublin, 20-950 Lublin, Poland*

[4] *Institute of Cell Biology, National Academy of Sciences of Ukraine, 79005 Lviv, Ukraine*

[5] *Department of Nuclear Chemistry, Comenius University in Bratislava, 84215 Bratislava, Slovakia*

[6] *Institute of Physics, Slovak Academy of Sciences, 84511 Bratislava, Slovakia*

[7] *Polymer Institute, Slovak Academy of Sciences, 84541 Bratislava, Slovakia*

*Corresponding author. E-mail address: osmutok@clarkson.edu (O. Smutok); ekatz@clarkson.edu (E. Katz)

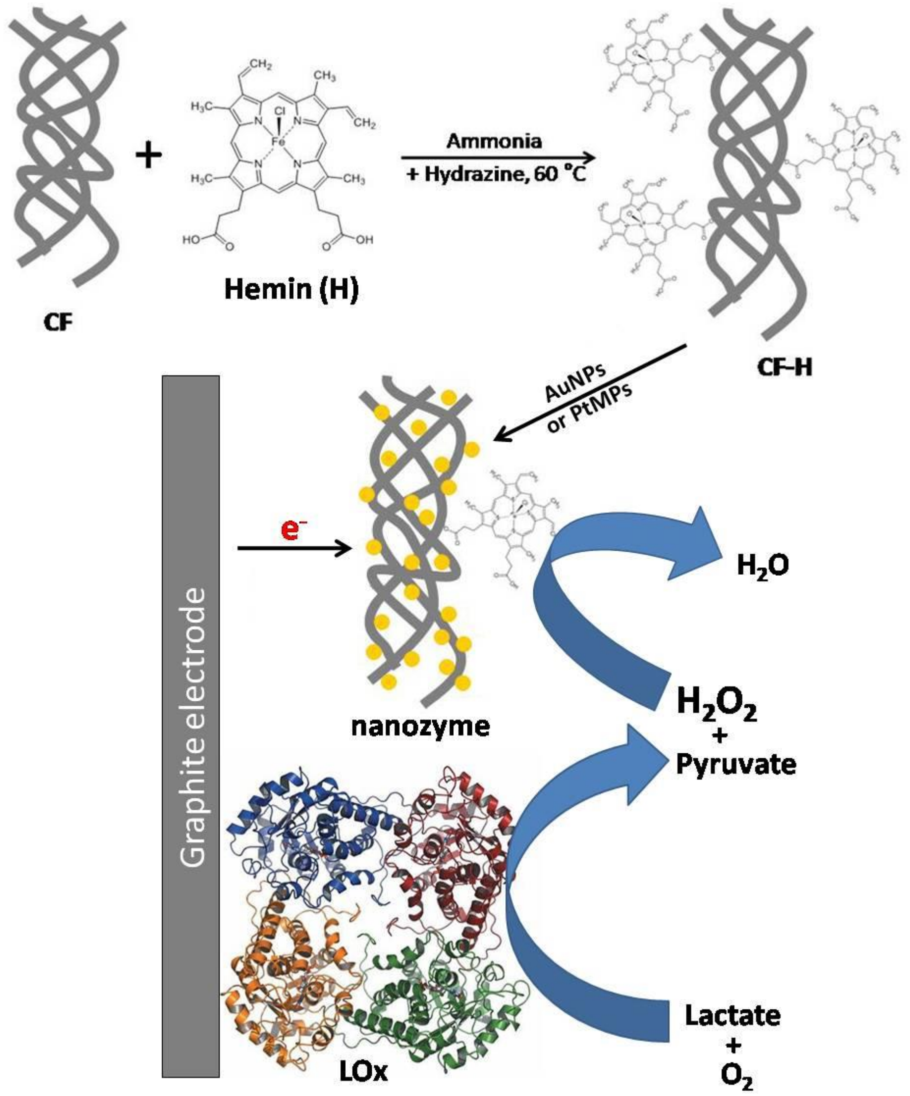

Figure S1. The schematic figure illustrates the research concept. Abbreviations: CF – carbon microfibers; H – hemin; CF-H – carbon microfibers modified by hemin; AuNPs – gold nanoparticles; PtMPs – platinum microparticles; LOx – lactate oxidase.

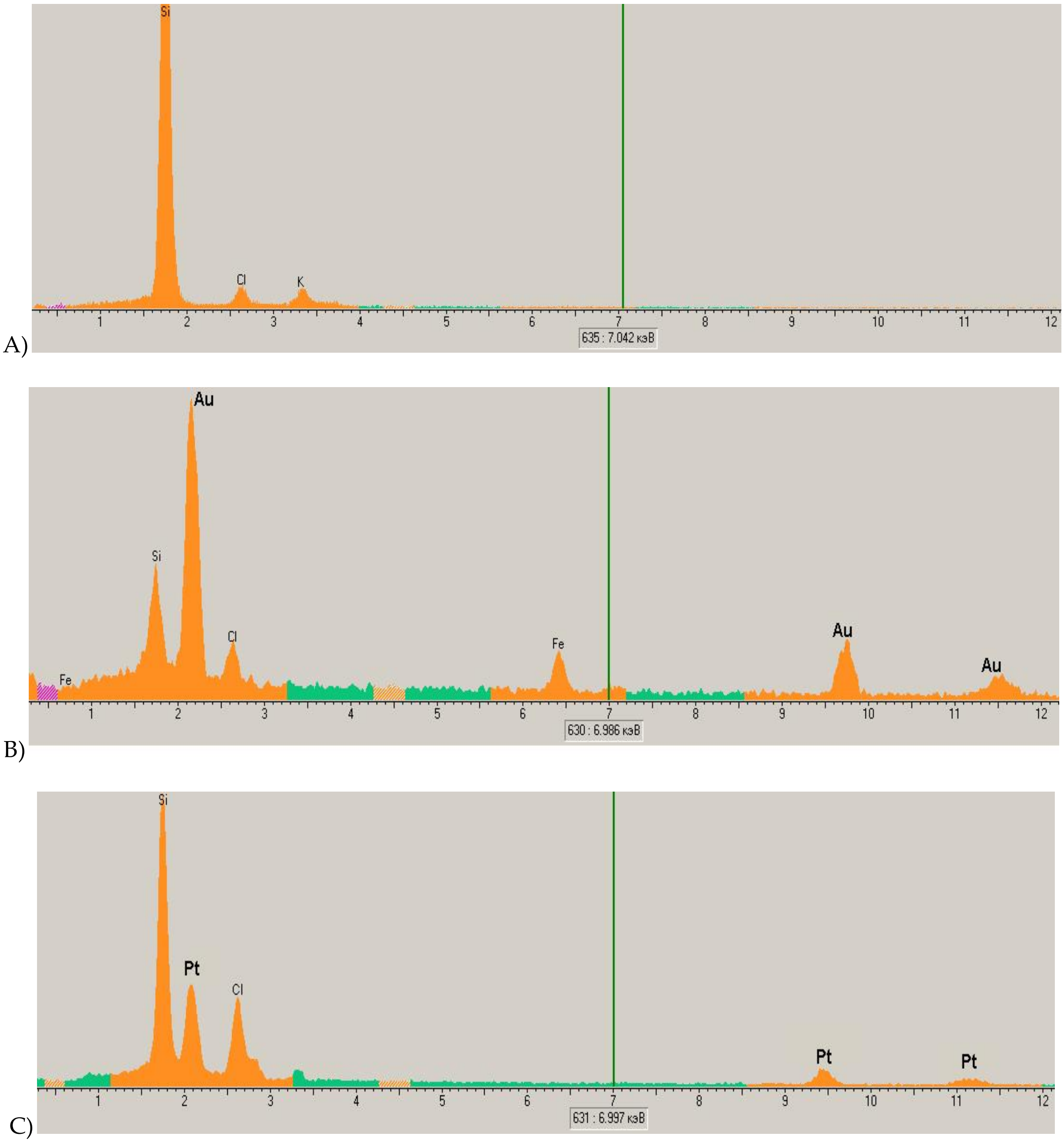

A)
B)
C)

Figure S2. X-ray spectral analysis of non-modified debris of CF (A), CF-H-AuNPs (B), and CF-H-PtMPs (C).

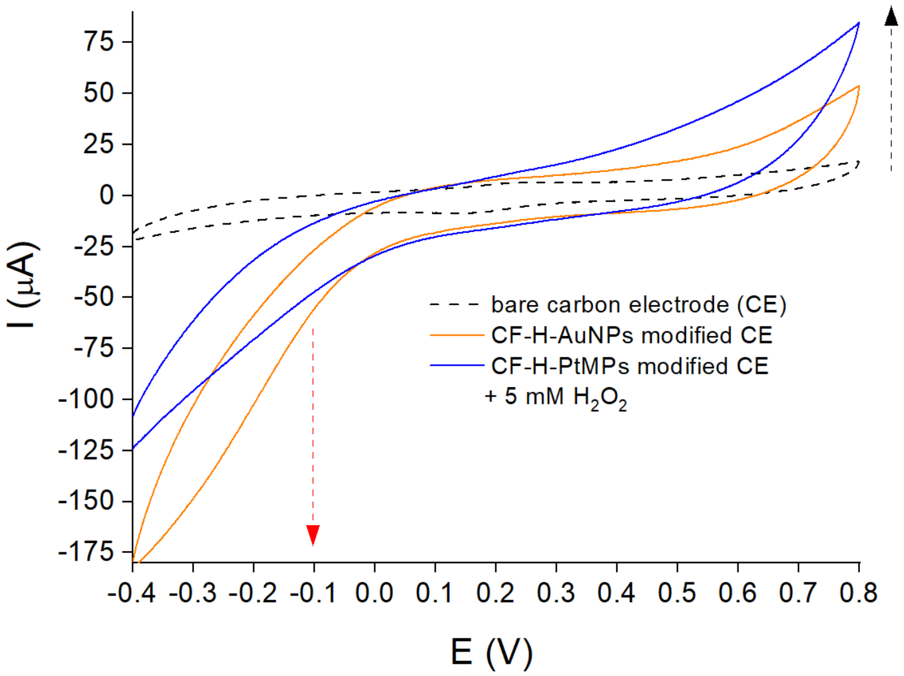

Figure S3. Cyclic voltamperogramms of bare graphite electrode (dash line), CF-H-AuNPs-modified (orange line), and CF-H-PtMPs-modified (blue line) electrodes at addition of 5 mM H_2O_2. Conditions: scan rate 50 mV·s^{-1} vs Ag/AgCl/KCl (3M) in 50 mM PB, pH 6.8 at room temperature.
